# Clinicopathologic and proteomic characteristics of low-grade undifferentiated spindle cell sarcoma

**DOI:** 10.3389/fmolb.2025.1591644

**Published:** 2025-07-01

**Authors:** Yani Wei, Hongjun Li, Shujin He, Min Li, Yongyu Chen, Huijuan Shi, Anjia Han

**Affiliations:** Department of Pathology, The First Affiliated Hospital, Sun Yat-sen University, Guangzhou, Guangdong, China

**Keywords:** undifferentiated spindle sarcoma, soft tissue tumor, proteomic characteristic, pathology, biomarker, diagnosis, molecular diagnosis

## Abstract

**Introduction:**

Undifferentiated spindle cell sarcoma (USCS) is a rare and heterogeneous group without specific diagnostic, prognostic, or predictive markers. The clinicopathologic and proteomic characteristics of USCS remain largely unknown.

**Methods:**

Between 2008 and 2024, we collected 14 low-grade USCSs and 104 undifferentiated pleomorphic sarcomas (UPSs). We conducted a comprehensive mass spectrometry (MS) proteomic analysis on USCSs and compared the clinicopathologic characteristics of low-grade USCSs and UPSs. More than 5600 proteins could be identified.

**Results:**

Low-grade USCSs had 353 upregulated and 500 downregulated proteins compared to corresponding normal tissue. PHRF1, DIDO1, RAPH1, GGT7, and PHF14 exhibited overexpression in low-grade USCSs, whereas SERPINF2, TMEM40, FYCO1, COL2A1, and NPNT demonstrated low expression. The KEGG pathway enrichment analysis revealed that most of the enriched pathways in low-grade USCS were related to various amino acid and lipid metabolic. Correlating significantly changed proteins with their targeting medications revealed novel therapy options for low-grade USCSs. Furthermore, in comparison to UPSs, our findings indicate that low-grade USCSs may exhibit smaller sizes and a lower rate of distant metastasis. In summary, to the best of our knowledge, this is the first in-depth proteomic analysis to demonstrate a comprehensive investigation of the clinicopathological and proteomic characteristics of low-grade USCSs.

**Conclusion:**

We initially elucidated the characteristics of differential proteins, the pathways enriched, and their possible drug targets in low-grade USCSs. Data are available via ProteomeXchange with identifier PXD061644.

## 1 Introduction

Undifferentiated soft tissue sarcoma (USTS) is a heterogeneous entity and shows no identifiable line of differentiation. USTSs can be categorized into pleomorphic, spindle cell, round cell, and epithelioid subsets. However, they lack distinct defining characteristics aside from their lack of identifiable differentiation (Fletcher, 2014; Antonescu and Organization, 2020). The treatment and prognosis for USTS with varying molecular characteristics, and research on USTS is still limited at now (Antonescu and Organization, 2020). Until recently, the genetics of USTS has been poorly characterized, primarily relying on small case series and low-resolution techniques, including karyotyping and array comparative genomic hybridization (Aspberg et al., 1995; Kresse et al., 2010).

Currently, with the development of the massive parallel sequencing and genome wide multi-omics, the genomic complexity and heterogeneity of USTS are becoming obvious (Steele et al., 2019). Whole exome and whole genome sequencing efforts have demonstrated differences in mutational frequencies, identified novel cancer driver gene mutations in USTS, and proposed new methods for classifying genomic instability in these tumors (2017; Steele et al., 2019). Firstly, the integration of accumulating genetic, epigenetic and transcriptomic data with emerging clinicopathological information and experimental models (Pierron et al., 2012; Antonescu et al., 2017; Koelsche et al., 2018; Watson et al., 2018; Koelsche et al., 2019) culminated in the inclusion of new chapter of ‘undifferentiated small round cell sarcomas (USRCSs) of bone and soft tissue’ in the 2020 WHO classification of tumor of soft and bone (Antonescu and Organization, 2020; Kallen and Hornick, 2021; Sbaraglia et al., 2021). Some studies have focused on investigating the characteristics of round cell undifferentiated sarcomas, demonstrating that these tumors can be categorized into Ewing sarcoma, round cell sarcoma with *EWSR1*-non-*ETS* fusions, *CIC*-rearranged sarcoma, and sarcoma with *BCOR* genetic alterations (Fletcher, 2014). Secondly, several studies have focused on the molecular characteristics of the undifferentiated pleomorphic sarcoma (UPS). The molecular data of pan-sarcoma molecular analysis based on 44 UPSs indicated that the UPSs and myxofibrosarcomas (MFSs) are not distinct entities, but rather fall along a spectrum, as in the original nomenclature for these tumors. A *YAP1/VGLL3* target gene signature was strongly expressed in UPS/MFS (Abeshouse et al., 2017). Another study has showed that the most significantly mutated genes in UPSs are *TP53*, *ATRX*, and *RB1*, while the most significantly mutated pathways are PI3K, TP53, and EPI (Nacev et al., 2022). However, there are still many bottlenecks that need to be addressed foremost amongst them are consistent criteria for the diagnosis of undifferentiated spindle cell sarcoma (USCS).

Studies on USCS are limited, primarily consisting of case reports, with no large number of cases reported. Consequently, the clinicopathologic and molecular characteristics of USCS remain largely unexplored. A fraction of tumors remains unclassifiable due to their lack of unique morphology and immunohistochemical characteristics, leading to the classification of unclassified mesenchymal neoplasms. The USTS category is decreasing at accelerated pace due to the application of gene and protein sequencing technologies. As a result, several new entities of spindle cell tumors with monomorphic cytomorphology have recently been identified, including *NTRK* and other kinase-fusion positive spindle cell tumors (Chiang et al., 2018; Suurmeijer et al., 2019; Kao et al., 2020), undifferentiated sarcomas with *BCOR-CCNB3* (Argani et al., 2017; Kao et al., 2018), *MEIS1-NCOA2/1* (Argani et al., 2018; Kao et al., 2021), *FUS-NACC*1 (Rooper et al., 2023), *EWSR1-NACC1* (Antonescu et al., 2021), *FGFR1-TACC1* (Yau et al., 2022), and *MEIS1-NCOA2* rearrangement (Coty-Fattal et al., 2024).

This study aimed to analyze the clinicopathologic and proteomic features of low-grade USCSs, to better understand the clinicopathologic and proteomics features of USCSs, and further to identify proteins that may serve as potential immunohistochemical markers for precise diagnosis.

## 2 Materials and methods

### 2.1 Sample selection

The consultation and surgical pathology files of the First Affiliated Hospital of Sun Yat-Sen University were searched for the diagnosis of undifferentiated sarcoma from January 2008 to May 2024. Hematoxylin and eosin-stained slides and previously performed immunohistochemical stains and molecular studies were reviewed in all cases retrieved. Clinicopathologic parameters, such as clinical history, age, gender, tumor location, tumor size, and follow-up information, were extracted from pathologic reports and electronic medical records. All cases in this study were re-evaluated by two pathologists and specialists in the soft tissue subspecialty of our department. They proposed potential differential diagnoses based on clinical information and morphology, and we conducted relevant immunohistochemical and molecular tests corresponding to the differential diagnoses for each case.

The inclusion criteria of USCS were patients 1) diagnosed as primary USCS, 2) who underwent surgery at our hospital, 3) possessing well-preserved surgical specimens, and 4) without prior chemoradiotherapy. The exclusion criteria of USCS were patients 1) with recurrent or metastatic USCS, 2) lack of postoperative specimens, 3) having poor-quality surgical specimens, and 4) those who received preoperative chemoradiotherapy.

The inclusion criteria of UPS were patients 1) diagnosed as primary UPS, 2) who underwent surgery at our hospital and 3) without prior chemoradiotherapy. The exclusion criteria of UPS were patients 1) with recurrent or metastatic UPS, 2) lack of postoperative specimens, and 3) those who received chemoradiotherapy.

After histological evaluation, 14 cases of USCS collected surgical specimen samples were eligible for subsequent mass spectrometry (MS), and 104 cases of UPS were used to perform statistical analysis. Approval for the use of formalin-fixed paraffin-embedded (FFPE) tissue for the study was obtained from the Ethics Review Committee of the First Affiliated Hospital of Sun Yat-sen University (2024-543).

### 2.2 Sample preparation

In this study, MS analyses were all based on postoperatively resected USCS FFPE samples. All tumors originated from mesenchyme and remained unaltered by decalcification. Samples were dewaxed with xylene. Subsequently, four volumes of lysis buffer (1% SDS, 1% protease inhibitor cocktail) were added to the tissue, followed by 3 minutes of sonication on ice using a high-intensity ultrasonic processor (Scientz). The remaining debris was eliminated through centrifugation at 12,000 g for 10 min at 4°C. The supernatant was collected, and protein concentration was measured using the BCA kit following the instructions.

The protein solution was prepared for digestion by reduction with 5 mM dithiothreitol for 30 min at 56°C, followed by alkylation with 11 mM iodoacetamide for 15 min at room temperature in the absence of light. The protein sample was subsequently diluted by incorporating 200 mM TEAB to acquire a urea concentration within 2 M. Trypsin was added at a 1:50 trypsin-to-protein mass ratio for the initial overnight digestion and at a 1:100 trypsin-to-protein mass ratio for a subsequent 4-h digestion. Finally, the peptides were desalted by Strata X SPE column.

### 2.3 Mass spectrometry

The peptides were solubilized using liquid chromatography mobile phase A and subsequently separated with an EASY-nLC 1, 200 ultra-high performance liquid chromatography (UPLC) system (ThermoFisher Scientific). The mobile phase consisted of solvent A (0.1% formic acid, 2% acetonitrile/in water) and solvent B (0.1% formic acid, 90% acetonitrile/in water). Peptides were separated with the following gradient: 0–68 min, 6%–23% B; 68–82 min, 23%–32% B; 82–86 min, 32%–80% B; 86–90 min, 80% B, and all at a constant flow rate of 500 nL/min. The peptides were separated using the UPLC system and subsequently injected into a NanoSpray Ionization (NSI) ion source for ionization, followed by analysis in the Orbitrap Exploris 480 equipped with a nano-electrospray ion source. The electrospray voltage applied was 2,300 V. Field Asymmetric Ion Mobility Spectrometry (FAIMS) compensate voltage (CV) was set as −45 V, −65 V. Precursors and fragments were analyzed at the Orbitrap detector. The full MS scan resolution was set to 60,000 for a scan range of 400–1, 200 m/z. The MS scan was fixed first mass as 110 m/z at a resolution of 15,000 with the TurboTMT was set as off. The data acquisition mode employed a data-dependent acquisition (DDA) procedure, in which after the primary scan, pre-peptide parent ions exhibiting the highest signal intensities were selected to sequentially enter the higher-energy collisional dissociation (HCD) collision cell for fragmentation at 27% energy, followed by the same sequential secondary MS analyses. To enhance the efficient use of MS, the automatic gain control (AGC) was set at 100% with an intensity threshold of 50,000 ions/s and a maximum injection time of Auto, and the dynamic exclusion time for tandem MS scans was fixed at 20 s to prevent redundant scans of the parent ions.

### 2.4 Database search

The resulting MS data were analyzed by the Proteome Discoverer search engine (v.2.4). Tandem mass spectra were searched against the Homo_sapiens_9606_SP_20230103.fasta database (20,389 entries), which was concatenated with a reverse decoy and contaminants database. Trypsin (Full) was designated as the cleavage enzyme, permitting up to 2 absent cleavages. We set the minimum peptide length to 6 and the maximum number of modifications per peptide to 3. We set the mass error to 10 ppm for precursor ions and 0.02 Da for-fragment ions and then specified carbamidomethyl on cysteine as a fixed modification. We specified oxidation of methionine, acetylation at the protein N-terminus, methionine loss, and methionine loss with concurrent acetylation as variable modifications. The false discovery rate (FDR) for proteins, peptides, and peptide-spectrum matches (PSMs) was controlled to be less than 1%.

### 2.5 Statistical analyses

Using the raw files of MS, we make a database of sample-specific proteins based on where the samples came from and then use analysis software to search the database. We conduct quality control analysis at the peptide and protein levels based on the database search results. We also conduct a quantitative analysis of the proteins, which should include a quantitative distribution analysis and a reproducibility analysis. Simultaneously, demonstrate the distribution of the sample’s quantitative intensity values. We use common functional annotations on the proteins that have been found, like TF annotations, COG/KOG, KEGG analysis, Reactome, WikiPathways, HallMark, and other protein domains and GO enrichment analyses. We used the quantitative results to figure out the fold change (FC) and the T-test significance P-value. We then sorted the differences by the thresholds and made the necessary statistical diagrams for difference analysis. We utilized statistics to categorize the functions of proteins that differed between the two groups. First, there was the GO secondary classification. Next, there was the subcellular localization classification. Finally, there was the COG/KOG classification. Fisher’s exact test enriched and analyzed differential proteins between the two groups. The related methods involve functional analyses of GO, KEGG, protein domains, Reactome, and WikiPathways. Protein-protein interaction (PPI) analysis was used to find the key proteins in each group based on the experiments. We also used DrugBank to predict the drug targets. The Supplementary Material provided detailed bioinformatics methods. To reduce the influence of confounding factors such as age, gender, and location on our results, we constructed linear mixed effects models (LMEs) and generalized linear models (GLIMs), which included the following covariates: Age: the age of the patient; Gender: the gender of the patient; Type: tissue type (tumor or normal); Patient: individual random effect of the patient and Location: anatomical site.

## 3 Results

### 3.1 Clinicopathologic features of the low-grade USCSs

**FIGURE 1:**
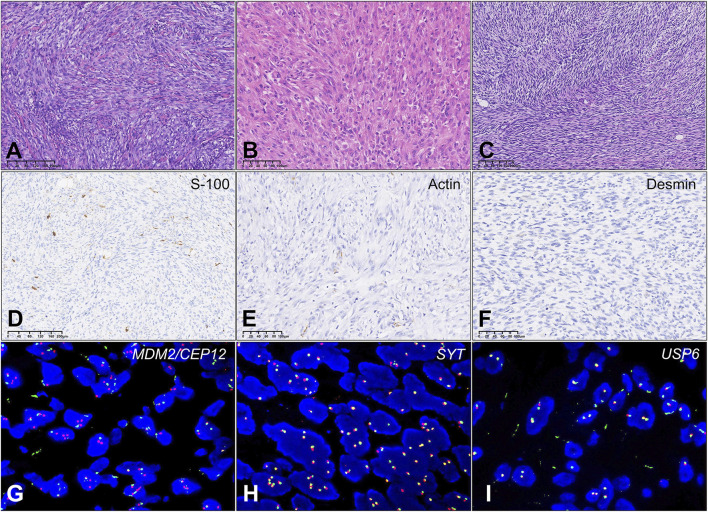
Results of differential protein screening in low-grade USCS and corresponding normal tissues. **(A)** Case T8a exhibits a significant presence of spindle cell hyperplasia characterized by storiform growth pattern, increased abundance of cell cytoplasm, and infiltration of inflammatory cells. Areas with wiry collagen and small vessels between tumor cells were also noted. **(B)** Case T10a Tumor tissue comprises ovoid, short, spindle-shaped atypical cells organized in fascicular patterns. The tumor cells had a more epithelioid appearance with abundant pale eosinophilic cytoplasm and syncytial borders. **(C)** Case T11a: Tumor showed a predominant storiform growth pattern composed of monomorphic spindle cells with delicate cell process. Focal area with collagen and small vessels were found. **(D)** Case T8b: Actin protein negativity. **(E)** Case T11a: Desmin protein negativity. **(F)** Case T10b: Focal S100 protein positivity. **(G)** Case T9a: MDM2/CEP12 negativity. **(H)** Case T11a: SYT negativity. **(I)** Case T8a: USP6 negativity.

Between 2008 and 2024, 14 cases with low-grade USCSs and 104 patients with UPSs were analyzed in this study. Representative cases of USCS are shown in Figures 1A–C. The clinicopathological characteristics, molecular testing results, and prognostic information of the 14 low-grade USCS patients were shown in Table 1. For low grade USCSs, there were 9 men and 5 women with median age of 59.5 years (range, 12–77 years). All patients presented with primary disease. Four cases (28.6%) occurred in the calf, two cases (14.3%) in the groin, two cases (14.3%) in the shoulder and back, and additional cases were noted in the ankle (7.1%), salivary gland (7.1%), retroperitoneum (7.1%), pelvis (7.1%), forearm (7.1%), and liver (7.1%).

**TABLE 1:** Summary of the baseline clinicopathologic characteristics of the low-grade undifferentiated spindle sarcoma.

No	Age	Gender	Anatomical location	Immunohistochemistry	Molecular testing	Follow up
T1	29	M	tibia	CK (+, focal), Vimentin (+), INI-1 (+), CD34 (+, partial), FLI-1 (−), EMA (+, partial, weak), S-100 (+, focal, weak), SOX10 (−), Ki-67 (20%+), MyoD1 (−), Myogenin (−), TL1-1 (+, focal), SATB2 (−), Desmin (−)	ND	20 (NED)
T2	77	M	saliva gland	CK (−), CK19 (−), EMA (−), CD99 (−), CD34 (−), Bcl-2 (+, focal), STAT-6 (−), Actin (−), Calponin (−), Desmin (−), MyoD1 (−), Myogenin (−), CD117 (−), DOG-1 (−), S-100 (−), Ki-67 (50%+)	*SYT* (−)	50 (NED)
T3	32	F	calf	Vimentin (+), H-Caldesmon (+, partial, weak), CD99 (+), CK (−), Actin (−), Desmin (−), CD31 (−), Calponin (−), S-100 (−), EMA (−), Bcl-2 (−), CD34 (−), CD68 (−), CD163 (−), CD10 (−), Ki-67 (10%+), P16 (−), CDK4 (−)	MDM2/CEP12 (−)	28 (NED)
T4	61	F	retroperitoneal	CD34 (−), Actin (−), S-100 (−), EMA (−), ALK (−), ER (−), PR (−), HMB-45 (−), Melan-A (−), STAT-6 (−), Desmin (−), MyoD1 (−), Myogenin (−), CD21 (−), CD23 (−), CD68 (+), CK (−), H3K27Me3 (expression)	*MDM2/CEP12* (−), *EWSR1* (−)	NA
T5	12	F	abdominal and pelvic	Myogenin (−), MyoD1 (−), ALK (D5F3) (−), ALK (D5F3)-Neg (−), CD30 (+), NTRK (−), Brg-1(SMARCA4) (expression), CD117 (−), DOG-1 (−), SDHB (expression), ALK (ALK1) (−), H-Caldesmon (−), Actin (−), Desmin (−), HMB-45 (−), Melan-A (−)	ND	22 (NED)
T6	37	F	scapula	FLI-1 (+, focal), CD31 (−), CD34 (−), Actin (−), Desmin (−), MyoD1 (−), Myogenin (−), S-100 (−), Ki-67 (10%+), RB (expression), P16 (−), CDK4 (−), CK (−), P63 (−)	ND	67 (NED)
T7	56	M	liver	CD117 (−), CD34 (−), DOG-1 (−), SDHB (+, weak), Actin (+, partial), Desmin (−), S-100 (−), Ki-67 (10%), H-Caldesmon (−), ALK (D5F3) (−), ALK (D5F3)-Neg (−), NTRK (−), STAT-6 (−), HMB-45 (−), Melan-A (−)	*SYT* (−)	3 (NED)
T8a	72	M	calf	Actin (+), CD34 (−), INI-1 (expression), Desmin (−), CD31 (−), ERG (−), S-100 (−), CD68 (−), CD163 (−), Ki-67 (10%–20%+)	*USP6* (−)	NA
T8b	72	M	calf	Actin, Desmin (−), MyoD1 (−), Myogenin (−), S-100 (−), CD34 (−), SOX10 (−), P53 (40%+), Ki-67 (40%+)	ND	NA
T9a	66	M	groin	P16 (+), CDK4 (+, focal), Actin (+, partial), Desmin (−), S-100 (−)	*MDM2/CEP12* (−), *CHOP* (−)	7 (NED)
T9b	61	M	shoulder and back	CD34 (−), Actin (+, partial), S-100 (−), EMA (−), Myogenin (−), MyoD1 (−), CK (−), INI-1 (expression), H3K27Me3 (expression)	ND	25 (NED)
T10a	76	F	forearm	HMB-45 (−), Melan-A (−), ALK (ALK1) (−), NTRK (−), P16 (−), CDK4 (+, partial), ERG (−), STAT-6 (−), H3K27Me3 (expression), Brg-1(SMARCA4) (expression), INI-1 (expression)	*MDM2/CEP12* (−)	60 (LR)
T10b	48	M	groin	CD34 (−), Desmin (−), Actin (+, partial), Calponin (+), H3K27Me3 (expression), S-100 (+, focal), SOX-10 (−), Ki-67 (40%+)	ND	3 (AM)
T11a	58	F	ankle	Actin (−), Desmin (−), S-100 (+, partial, weak), SOX10 (−), H3K27Me3 (expression), P16 (−), CDK4 (−), CD34 (+, partial), STAT-6 (−), MyoD1 (−), Myogenin (−), Ki-67 (20%+)	*SYT* (−)	4 (NED)

NA, not available; ND, not done; NED, no evidence of disease; AM, abdomen metastasis; LR, local recurrence.

The immunohistochemistry staining of 14 low-grade USCS patients was: 1) 12 patients (12/14, 85.7%) performed S-100 tests; 9 were negative (9/12, 75.0%), and 3 (3/12, 25.0%) were focal or partial weak positive (Figure 1D). 2) Actin was conducted on 11 patients (11/14, 78.6%), 8 of whom were negative (8/11, 72.7%) and 3 (3/11, 27.3%) partial or positive (Figure 1E). 3) 11 patients (11/14, 78.6%) performed CD34 tests, 10 of which were negative (10/11, 90.9%) and 1 (1/11, 9.1%) partially positive. 4) All 10 patients (10/14, 71.4%) tested negative for Desmin (Figure 1F). 5) All 7 patients (7/14, 50%) were negative for MyoD1 and Myogenin.

The results of the fluorescence *in situ* hybridization (FISH) analysis, which include *SYT*, *MDM2/CEP12* (Figure 1G), *EWSR1*, *SYT* (Figure 1H), *USP6* (Figure 1I), and *CHOP*, were negative. Local recurrence was observed 60 months post-surgery in one case, while abdominal metastasis was noted 3 months post-surgery in another case.

### 3.2 Comparison of the characteristics in low-grade USCSs and UPSs

The baseline characteristics and comparison results of the low-grade USCSs and UPSs were shown in Table 2. For UPSs, there were 66 men and 38 women with median age of 55.0 years (range, 13–91 years). All patients presented with primary disease. Sixty-four cases (64/104, 61.5%) were located in the lower limb, twelve cases (12/104, 11.5%) in the upper limb, twelve cases (12/104, 11.5%) in the abdomen, five cases (5/104, 4.8%) in the back, four cases (4/104, 3.8%) in the chest, two cases (2/104, 1.9%) in the head and neck, and additional cases were noted in the liver (1/104, 1.0%), kidney (1/104, 1.0%), axilla (1/104, 1.0%), and supraclavicular fossa (1/104, 1.0%).

**TABLE 2:** Comparison of the clinicopathological features in low-grade USCS and UPS patients.

Features	Total (N = 118)	Low-grade USCS (N = 14)	UPS (N = 104)	*P* USCS vs. UPS
Age				
Mean	54.2 ± 16.4	54.1 ± 19.8	54.22 ± 16.0	0.975
≤50y	46 (39.0)	5 (35.7)	41 (39.4)	0.789
>50y	72 (61.0)	9 (64.3)	63 (60.6)	
Gender				
Female	43 (36.4)	5 (35.7) 5	38 (36.5)	0.952
Male	75 (63.6)	9 (64.3)	66 (63.5)	
Tumor size				
Mean (cm)	9.1 ± 5.2	7.6 ± 6.5	9.3 ± 5.0	0.245
Location				
abdomen	16 (13.6)	4 (28.6)	12 (11.5)	0.398
axilla	1 (0.8)	0 (0.0)	1 (0.8)	
back	6 (5.1)	1 (7.1)	5 (4.8)	
chest	5 (4.2)	0 (0.0)	5 (4.2)	
head and neck	3 (2.5)	1 (7.1)	2 (1.9)	
kidney	1 (0.8)	0 (0.0)	1 (0.8)	
liver	2 (1.7)	1 (0.8)	1 (0.8)	
lower limb	69 (58.5)	5 (35.7)	64 (61.5)	
supraclavicular fossa	1 (0.8)	0 (0.0)	1 (1.0)	
upper limb	14 (11.9)	2 (14.3)	12 (11.5)	
Distant metastasis				
Yes	16 (13.6)	1 (7.1)	15 (14.4)	0.681
No	69 (58.5)	10 (71.4)	59 (56.7)	
NA	33 (28.0)	3 (21.4)	30 (28.8)	
Mean (cm)	9.1 ± 5.2	7.6 ± 6.5	9.3 ± 5.0	

NA, not available.

The comparative results presented in our study indicated no statistically significant differences in the clinicopathological characteristics of low-grade USCS and UPS, including age (*P* = 0.975), gender (*P* = 0.952), tumor size (*P* = 0.245), location (*P* = 0.398), and the presence of distant metastases (*P* = 0.922). Despite the lack of statistically significant differences in the comparisons, likely attributable to the limited data, it was evident that the tumor size of UPSs was greater than that of low-grade USCSs (9.3 cm vs. 7.6 cm, *P* = 0.245), the occurrence probability in the lower limbs was higher for UPSs compared to USCSs (61.5% vs. 35.7%, *P* = 0.398), and the rate of distant metastasis was higher for UPSs than for low-grade USCSs (14.4% vs. 7.1%, *P* = 0.681). Nevertheless, the disparities between the two entities require an increased sample size and further investigation.

### 3.3 Mass spectrometry-based proteomics on 14 low-grade USCSs

#### 3.3.1 Data quality control and Basic analysis

A total of 6,677 proteins were identified, with 5,687 proteins available for comparison (Supplementary Table S1). The distribution of peptide lengths showed that the distribution of peptide lengths identified by MS in this study met the quality control requirements (Supplementary Figure S1A). The distribution map of peptide segments indicated that most proteins were paired with multiple peptides (Supplementary Figure S1B). The protein coverage distribution map demonstrated that many proteins exhibit coverage of less than 30% (Supplementary Figure S1C). The protein molecular weight distribution graph indicated a uniform distribution of the identified proteins, suggesting that the experimental process did not lead to the loss of proteins within specific molecular weight ranges (Supplementary Figure S1D). The quantitative analysis results of the intensity values for each protein across the various samples were presented in Supplementary Tables S2A, S2B. The results of the repeatability analysis, which encompass Pearson’s Correlation Coefficient (PCC), Principal Component Analysis (PCA), and Relative Standard Deviation (RSD), are presented in Supplementary Figure S2. All three analyses indicated that the reproducibility of the grouped samples in this study fulfilled the primary requirements for MS analysis. The violin plots of intensity value distribution indicated that the sample means in this study were close to the same level, indicating good sample quality (Supplementary Figure S3).

#### 3.3.2 Protein profiling of low-grade USCSs

**FIGURE 2:**
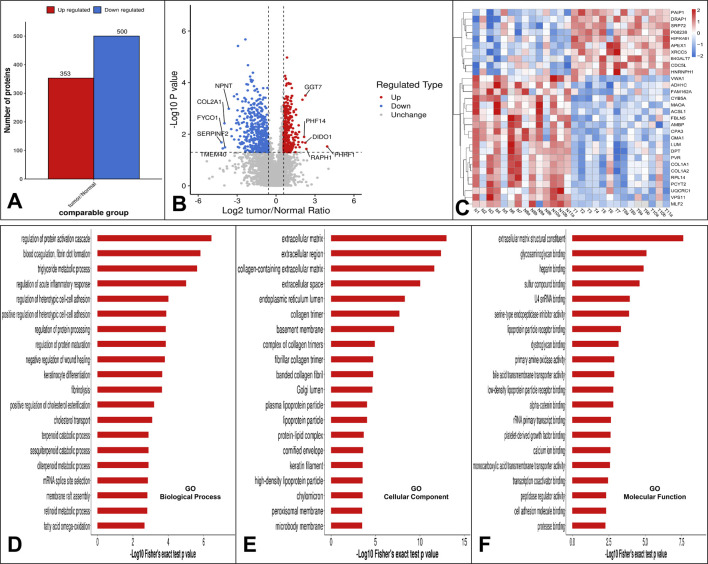
**(A)** Statistical results of differential protein analysis. Our results showed that 353 proteins were upregulated and 500 proteins were downregulated in low-grade USCS compared to corresponding normal tissues. **(B)** The top five upregulated proteins were PHRF1, RAPH1, DIDO1, GGT7, PHF14 and the top five downregulated proteins were SERPINF2, TMEM40, FYCO1, COL2A1 and NPNT. **(C)** Hierarchical cluster analysis heatmap of all 14 sarcomas. Cluster formation in the heatmap showed a clear separation of the low-grade USCSs and normal tissues. **(D)** GO function enrichment analysis. Enrichment bar graph for GO biological process. **(E)** Enrichment bar graph for GO cellular component. **(F)** Enrichment bar graph for GO molecular function. The enrichment bar chart displays the 20 most significantly enriched functions. The vertical axis presents descriptive information for the corresponding GO function, while the horizontal axis indicates the -Log10 transformed enrichment significance P value, where larger values denote greater enrichment significance.

To gain a thorough understanding of the functional properties of different proteins, we performed a full range of functional annotations on the identified proteins. The results of the differential protein screen were presented in Supplementary Table S3. Comparing the protein expression profile of low-grade USCSs and corresponding normal tissues, applying a significance threshold of P < 0.05 and a net log2-fold change of more than 1.5, our results showed that 353 proteins were upregulated and 500 proteins were downregulated in low-grade USCSs compared to the corresponding normal tissues (Figure 2A).

The top five upregulated proteins were PHRF1, RAPH1, DIDO1, GGT7, and PHF14 and the top five downregulated proteins were SERPINF2, TMEM40, FYCO1, COL2A1 and NPNT (Figure 2B). Using the top 30 statistically significant differentially expressed proteins among low-grade USCSs and normal tissues with a significance threshold of *P* < 0.05, we performed a hierarchical cluster analysis to generate a heatmap of all 14 sarcomas of our study cohort (Figure 2C). Cluster formation in the heatmap showed a clear separation of the low-grade USCSs and normal tissues. Based on the proteomic signature, it was observed that the low-grade USCS and normal tissues in two cases exhibited identical proteins: T1 and N1 shared the protein VWA1, while T2 and N2 shared the protein HNRNPH1. In contrast, the remaining 12 paired low-grade USCS and normal samples exhibited a distinct proteomic signature. LMEs and GLIMs analyses revealed significant differences in the expression of the GGT7 and DIDO1 by the GLIMs (Pr (>|t|) < 0.05). This indicates that the expression differences between tumor and normal tissues remained statistically significant after controlling for confounding factors, including age, gender, and site. The results of the LMEs and GLIMs analyses were shown in Table 3.

**TABLE 3:** LME and GLIM analysis results.

Protein	Estimate	Std. Error	t value	Pr (>|t|)	FDR
PHRF1	13.60991	7.628679	1.784046	0.14898	0.372451
RAPH1	−0.19527	1.673248	−0.1167	0.909971	0.961097
GGT7	5.849877	0.657358	8.899076	2.34E-06	2.34E-05
DIDO1	10.6748	3.992107	2.673976	0.019119	0.095595
PHF14	0.323489	0.415171	0.779171	0.492695	0.961097
IBA57	−0.38219	1.853945	−0.20615	0.843489	0.961097
FBXO2	−6.05527	2.741476	−2.20876	0.062912	0.209707
HMGN1	1.130802	3.720135	0.303968	0.768908	0.961097
IRF2BP1	−0.06678	1.340827	−0.0498	0.961097	0.961097
DDX39A	−0.15762	0.772589	−0.20402	0.84176	0.961097

LME, linear mixed effects model; GLIM, generalized linear model; Std. error, Standard Error; FDR, false discovery rate.

#### 3.3.3 Analysis of biological function enrichment utilizing proteomic data

To further elucidate biological function in the low-grade USCSs, we performed GO, KEGG, protein domain, Reactome and WikiPathways enrichment analyses based on upregulated and downregulated proteins in low-grade USCS and the paired normal tissues.

Top five enriched GO biological processes in low-grade USCSs were regulation of protein activation cascade, blood coagulation and fibrin clot formation, triglyceride metabolic process, regulation of acute inflammatory response, and regulation of heterotypic cell-cell adhesion (Figure 2D). Top five enriched cellular components in low-grade USCSs were extracellular matrix, extracellular region, collagen-containing extracellular matrix, extracellular space, and endoplasmic reticulum lumen (Figure 2E). Top five enriched molecular functions were extracellular matrix structural constituent, glycosaminoglycan binding, heparin binding, sulfur compound binding, and U4 small nuclear RNA binding (Figure 2F).

**FIGURE 3:**
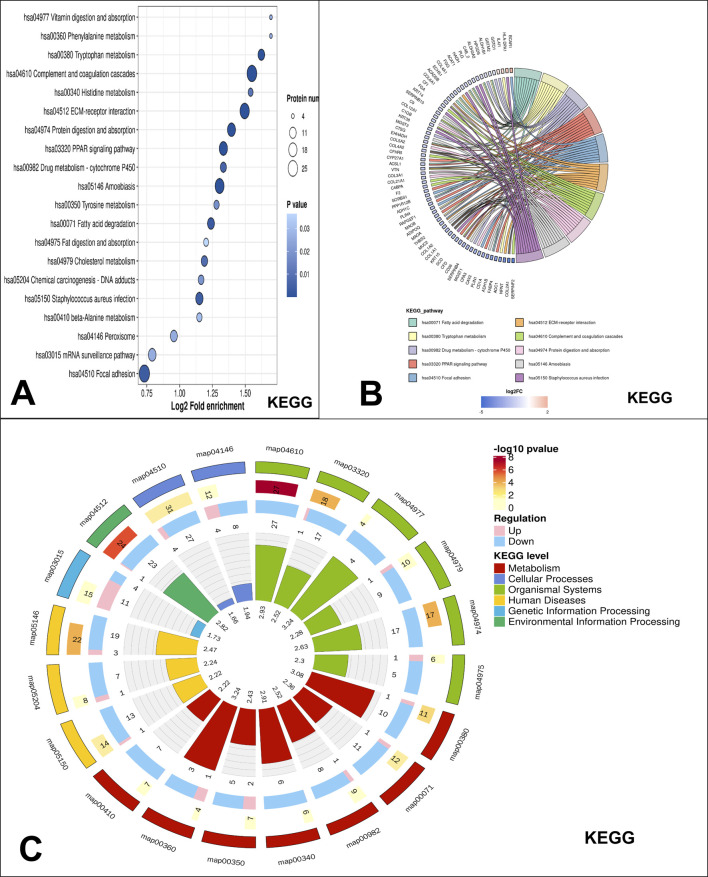
KEGG pathway enrichment analysis. **(A)** KEGG significantly enriched bubble map, the top enriched pathway in low-grade USCSs was the complement and coagulation cascades. **(B)** Significant enrichment of the chord diagram in KEGG. The proteins identified as enriched in the complement and coagulation cascades pathway include CFI, FGA, C9, C1QB, CFD, CFHR5, VTN, C4BPA, F2, and SERPINF2. **(C)** Significant enrichment of the circos plot in KEGG. The highest differential protein enrichment pathway was organismal systems and metabolism pathway.

In the KEGG enrichment analysis, the top enriched pathway in low-grade USCSs was the complement and coagulation cascades (Figure 3A). The proteins identified as enriched in the complement and coagulation cascades pathway include CFI, FGA, C9, C1QB, CFD, CFHR5, VTN, C4BPA, F2, and SERPINF2 (Figure 3B). The low-grade USCS Differential Protein enrichment circos plot indicated that the pathway categories demonstrating the highest differential protein enrichment were organismal systems, with 82 enriched proteins (3 upregulated and 79 downregulated), and metabolism, with 56 enriched proteins (6 upregulated and 50 downregulated) (Figure 3C).

This study included protein Domain, Reactome, and WikiPathways enrichment analyses, with results detailed in the Supplementary Material. The laminin EGF domain was identified as the most enriched Protein domain in low-grade USCSs (Supplementary Figure S4). The top enriched pathway identified in low-grade USCSs through Reactome enrichment analysis was extracellular matrix organization (Supplementary Figure S5). The top enriched pathway identified in low-grade USCSs in WikiPathways analysis was the miRNA targets in extracellular matrix and membrane receptors (Supplementary Figure S6).

### 3.4 Protein interaction network analysis

We identified the five proteins with the most significant interactions and delineated their interaction networks. The five most prominent proteins identified were CDK1, LBP, SNRPD3, HNRNPA2B1, and DRSF1. Detailed results were shown in Supplementary Table S4 and Supplementary Figure S7.

### 3.5 DrugBank drug target prediction results

**FIGURE 4:**
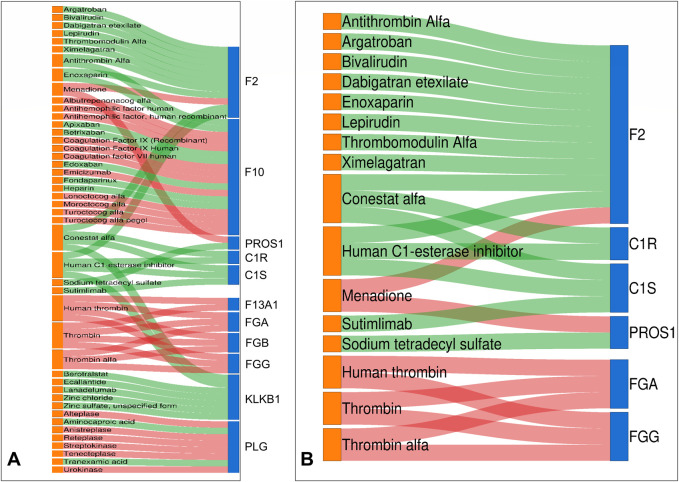
Drug-target Sankey diagram. Proteins identified as drug targets in both KEGG and GO include F2, PROS1, C1R, C1S, FGA, and FGG. **(A)** In the complement and coagulation cascades pathway of KEGG, we found 11 proteins with targeted drugs. **(B)** The function of the most enriched proteins in GO is the regulation of protein activation cascades. We identified six proteins associated with corresponding target drugs. Drug colors on the left show orange for false discovery rate-approved medications. Differential proteins on the right are indicated as follows: upregulated in red and downregulated in blue. The center line indicates the drug-target association: red denotes inhibitor, while green signifies activator.

We identified potential new therapeutic options in low-grade USCSs by correlating significantly altered proteins with their corresponding targeting drugs (Supplementary Table S5). In this study, we also predicted the protein targeting drugs in the function and pathway that were enriched by the largest number of proteins. Within the complement and coagulation cascades pathway of KEGG, we identified 11 proteins that currently had corresponding targeted drugs, including F2, F10, PROS1, C1R, C1S, F13A1, FGA, FGB, FGG, KLKB1, and PLG (Figure 4A). The function of the most enriched proteins in GO was the regulation of protein activation cascades. We identified six proteins associated with corresponding target drugs: F2, C1R, C1S, PROS1, FGA, and FGG (Figure 4B).

## 4 Discussion

Oncological practice MS-based proteomics utilizing FFPE tissue has become feasible given recent technology advancements (Coscia et al., 2020). In this study, we applied MS techniques to analyze the protein profile characteristics of 14 pairs of low-grade USCS and corresponding normal tissues, as well as the differences in clinicopathological characteristics between low-grade USCSs and UPSs. This study utilized shotgun MS-based data to quantitatively analyze over 5600 proteins, establishing the protein profile characteristics of low-grade USCS. By comparing and analyzing the clinicopathological characteristics of low-grade USCS with UPS, the study identified the differences of these two entities.

Our results showed that low-grade USCS was a heterogeneous group that could develop at a wide age (12–77 years), preferably in the lower leg, and its immunohistochemical markers and molecular tests are not specific. Our findings indicated that most USCS were negative for S-100, Actin, Desmin, CD34, MyoD1, and Myogenin. Cases 1, 10b, and 11a in the current investigation showed focal or partial positivity for S-100, and focal positivity for S-100 has also been documented in the literature in USCS with EWSR1/FUS-NACC1 fusions (Antonescu et al., 2021; Rooper et al., 2023), which is consistent with our findings. The majority of USCS cases are negative for S-100, with only a small percentage exhibiting positive S-100 results. Consequently, the presence of S-100 may indicate changes in specific molecules within USCS; however, the relevance of S-100 in this entity requires further validation. The existing literature on USCS is primarily comprised of case reports (Antonescu et al., 2021; Rooper et al., 2023; Coty-Fattal et al., 2024), indicating a need for further accumulation of cases to enhance the understanding of immunohistochemical marker expression in USCS.

USTS shows no identifiable line of differentiation, including USCS, UPS, and USRCS (Antonescu and Organization, 2020; Kallen and Hornick, 2021; Sbaraglia et al., 2021). Current research indicates that USRCS can be further categorized into Ewing sarcoma, URCS with *EWSR1*-non-*ETS* fusions, *CIC*-rearranged sarcoma, and sarcoma with *BCOR* genetic alterations (Cidre-Aranaz et al., 2022). Furthermore, a growing number of research are focusing on the clinical and molecular characteristics of UPS (Jibbe et al., 2022; Lu et al., 2024). The findings indicate that UPS and MFS exhibit significant similarities at the genomic, methylation, and transcriptomic levels, potentially categorizing them as a single disease entity differentiated solely by histologic phenotypes, such as the mucus matrix ratio. UPS is defined by a high prevalence of copy number variants (CNVs), a low somatic mutation burden, and recurrent mutations in a limited number of genes, such as *TP53*, *ATRX*, and *RB1*. Amplified genes of significance include *CCNE1* at 10%, *VGLL3* at 11%, and *YAP1* at 3% (Abeshouse et al., 2017). Another study showed that *COL6A3* was most predictive of overall survival in UPS patients and outperformed an established sarcoma prognostic gene panel at predicting metastasis in UPS (Klein et al., 2024). However, molecular studies of UPS have not yet addressed the proteomic features. Compared with the clinicopathological features of UPSs, low-grade USCSs were smaller in size and had a lower rate of distant metastasis. However, more study and a larger sample size are necessary to account for these differences. Due to the limited number of low-grade USCS cases, significant statistical differences were not observed in the analysis of the clinicopathological features of USCS and UPS. However, our study indicated potential differences between the two regarding tumor size and the rate of distant metastasis. UPS exhibited greater size and a higher tendency for distant metastasis, suggesting that the biological behavior of these tumors aligns with their histological characteristics. These findings may offer valuable reference information for pathologists in the diagnostic process. Comparative analysis of the clinicopathologic features of USCS and UPS revealed some differences, suggesting potential molecular distinctions between the two entities. Further clarification of the molecular features of UPS and exploration of the differences in clinical and molecular characteristics, including DNA, RNA, and proteomic features, between UPS and USCS could enhance the classification of USCS, which is essential in clinical practice.

Protein profiling identified 353 upregulated and 500 downregulated proteins in low-grade USCS compared to paired normal tissue samples. Among them, the top five upregulated proteins were PHRF1, RAPH1, DIDO1, GGT7, and PHF14. PHRF1 downregulation has been observed colorectal cancer (Lin et al., 2023), lung cancer (Wang et al., 2016) and leukemia (Prunier et al., 2015), alterations in PHRF1 expression facilitate the progression of the tumors. PHRF1 protein was upregulated in low-grade USCS compared to paired normal tissues, contrary to previous findings in leukemia, suggesting that its role in soft tissue sarcoma may be different from leukemia. It has been shown that RAPH1 promotes aggressiveness and radioresistance in breast cancer (Liu Q. et al., 2023) and that high expression of RAPH1 protein is associated with breast cancer shorter survival (Kurozumi et al., 2018). Loss of GGT7 may increase the cellular reactive oxygen species levels, inducing glioblastoma occurrence and growth (Bui et al., 2015). It has been reported that DIDO1 can be suggested as a marker for the primary esophageal squamous cell carcinomas (Forghanifard et al., 2020). PHF14 can promote lung adenocarcinoma metastasis (Tian et al., 2023) and promotes cell proliferation and migration in gastric cancer cells (Zhao et al., 2020). The top five downregulated proteins were SERPINF2, TMEM40, FYCO1, COL2A1, and NPNT. The proteins above have been up- or downregulated in other cancers, but not in the low-grade UCSC. SERPINF2 protein expression was upregulated in B-cell acute lymphoblastic leukemia (Cavalcante Mde et al., 2016) but downregulated in low-grade USCSs. High TMEM40 expression correlates with malignant behavior and tumorigenesis in bladder cancer (Zhang et al., 2018), tongue squamous cell carcinoma (Zhang et al., 2019), and cervical cancer (Zhang et al., 2023), whereas decreased TMEM40 expression is linked to malignant behavior in cutaneous squamous cell carcinoma (Yu et al., 2021), Recent studies suggest that COL2A1 may serve as a promising biomarker and therapeutic target for chondrosarcoma (Miwa et al., 2022). NPNT promotes early-stage bone metastases in breast cancer (Wang et al., 2018). The current role of the proteins in USCS remains may offer valuable insights for the precise diagnosis and treatment for USCS.

The KEGG pathway enrichment analysis revealed that the majority of the top 20 enriched pathways for proteins in low-grade USCS were related to metabolism. This included various amino acid metabolic pathways, such as those for tryptophan, histidine, and phenylalanine, as well as lipid and drug metabolism. The results in our study are consistent with existing studies in the literature that altered metabolic pathways in tumor cells are important drivers of tumorigenesis and progression (Cairns et al., 2011; Hanahan and Weinberg, 2011). The unique metabolic pathways of low-grade USCS relative to other tumor types require further investigation. Although many studies have focused on fatty acid metabolism (Liu P. S. et al., 2023), the enrichment of amino acid metabolism and drug metabolism in low-grade USCS may provide new insights into the unique characteristics of this tumor type.

Proteins identified as drug targets in both KEGG and GO include F2, PROS1, C1R, C1S, FGA, and FGG. We conducted a search for the specific drugs utilized in the clinic. Most of the targeted drugs have received FDA approval and are currently used in clinical practice. Conestat alfa (Plosker, 2012), Recombinant human C1 esterase inhibitor (Greve and Hahn, 2017), Berotralstat (Lee, 2021), Ecallantide (Garnock-Jones, 2010), Lanadelumab (Syed, 2019) and Sodium tetradecyl sulfate (Jenkinson et al., 2017) can treat vascular-related diseases. Argatroban inhibits breast cancer metastasis to bone (Asanuma et al., 2013). Bivalirudin reduces platelet and monocyte activation after elective percutaneous coronary intervention (Busch et al., 2009). Thrombomodulin Alfa for Acute Exacerbation of Idiopathic Pulmonary Fibrosis (Kondoh et al., 2020). Sutimlimab (sutimlimab-jome; ENJAYMO™) is a humanized monoclonal antibody developed by Sanofi for the treatment of cold agglutinin disease (CAD) (Dhillon, 2022). The identification of these drugs may offer potential targets for precision treatment of USCS.

Our study has some limitations. First, the rarity of low-grade USCS restricts the sample size in this study, requiring additional validation of the MS protein profiling results by collecting more cases of this entity to identify the potential specific diagnostic marker for low-grade USCS. Second, since limited data have been reported on the proteomic characterization of other subtype of low-grade USTS, only the clinicopathological features of USCS and UPS could be compared and analyzed in this study. A future comparative analysis of protein profiles across various groups of undifferentiated sarcomas may help in the precise diagnosis of undifferentiated sarcomas.

In conclusion, to the best of our knowledge, this is the first in-depth proteomic analysis of low-grade USCS. This study presents a comprehensive investigation of the clinicopathological and proteomic characteristics of low-grade USCS and investigates the clinicopathologic characteristics of UPS and low-grade USCS. Low-grade USCSs might be smaller and have a lower distant metastatic rate than UPSs. Additionally, we initially elucidated the characteristics of differential proteins in low-grade USCS, the pathways enriched, and their possible drug targets by MS sequencing analysis of low-grade USCS and its paired normal tissues, and the identified differential proteins may become potential biomarkers for the precise diagnosis and treatment of low-grade USCS.

## Data Availability

The datasets presented in this study can be found in online repositories. The names of the repository/repositories and accession number(s) can be found below: https://www.ebi.ac.uk/pride/archive/projects/PXD061644.
