# Supplementary material for: Clinicopathologic and proteomic characteristics of low-grade undifferentiated spindle cell sarcoma
Source: Front Mol Biosci. 2025 Jul 1;12:1591644. doi: 10.3389/fmolb.2025.1591644 (PMC12259450; doi:10.3389/fmolb.2025.1591644)
Supplement: Supplementary file 3 [file Supplementaryfile1.docx]

Supplementary Material

# Supplementary Material and Methods

## Data quality control

Mass spectrometry (MS) analysis provides the mass-to-charge ratio and signal intensity of peptides within a sample, along with the mass-to-charge ratio and signal intensity of fragment ions following peptide fragmentation. The data pertaining to peptides is referred to as a primary spectrum, while the information regarding peptide fragmentation ions is termed a secondary spectrum. The complexity of the information in the spectra necessitates the use of a database to accurately resolve the potentially present peptide sequences. A database of theoretical secondary spectra is constructed from the protein sequences prior to conducting the search. The secondary spectrum produced by mass spectrometry is analysed and compared with the theoretical secondary spectrum, resulting in the identification of accurately matched theoretical peptide sequences through algorithmic scoring and filtering. Identifying protein-specific peptides allows for the recognition of the associated protein information.

Following the completion of the library search, the data obtained from the MS downlink requires a series of quality control assessments to verify that the results adhere to established standards. These assessments include evaluations of peptide length distribution, peptide number distribution, protein coverage distribution, and protein molecular weight distribution.

## Quantitative analysis

The intensity values LFQ intensity for each protein in the different samples are given in the search library results. The LFQ intensities (I) of the proteins in different samples were transformed by centering to obtain the relative quantitative values (R) of the proteins in different samples. The formula is expressed as follows: where i represents a sample and j represents a protein.

Rij=Iij/Mean (Ij)

## Sample Repeatability Analysis

It is essential to assess the statistical consistency of quantitative results from biologically or technically duplicated samples. This study employed three statistical analyses—Pearson's Correlation Coefficient (PCC), Principal Component Analysis (PCA), and Relative Standard Deviation (RSD)—to evaluate repeatability.

## Intensity value distribution

We extracted protein intensity values from various samples to analyze their distribution and variability, presenting the results as violin plots.

## Annotation Methods

To thoroughly understand the functional properties of different proteins, we performed a comprehensive functional annotation of the identified proteins. These include Gene Ontology (GO), Protein domain, KEGG pathway, KOG functional classification, Subcellular localization, Reactome, WikiPathways, HallMark, and Transcription factor (TF) annotations.

## 1.5.1 GO Annotation

Gene ontology (GO) analysis is a bioinformatics analysis method that provides statistical information by organically linking information about genes and gene products. Proteomics projects primarily use GO for three purposes: 1) as a database for various protein and gene information; 2) to provide various protein and gene information and classify them according to the information; and 3) as a tool to provide the most comprehensive information annotation and classification service for all proteins in the project. GO analysis mainly includes three aspects: 1) "Cellular component" refers to specific components of a cell, which are considered constituent parts of larger cellular structures within the GO system; 2) "Molecular function" primarily refers to the chemical activities of molecules at the molecular level, such as binding or catalytic activities; and 3) "Biological process" refers to the planned and specific way a group of molecules carry out a certain function inside an organism. The GO annotation process starts with using the eggnog-mapper software to get GO IDs from the proteins that have been identified in the EggNOG database. The proteins are then subjected to functional classification annotation analysis based on their roles in biological processes, molecular functions, and cellular components.

**1.5.2 Domain Annotation**

We performed protein structural domain annotation on the identified proteins in the project data, using the Protein families (Pfam) database and the corresponding PfamScan tool.

**1.5.3 KEGG Pathway Annotation**

The Kyoto Encyclopedia of Genes and Genomes (KEGG) integrates currently known protein-protein interaction network information, such as pathways and related complexes (Pathway database), genes and gene products (Gene database) and biological complexes and related reactions (Compound and Reaction databases). We use the KEGG pathway database to annotate protein pathways and the BLAST comparison (blastp, evalue ≤ 1e-4) to find proteins. For each sequence, the annotation is based on the best-scoring comparison result.

**1.5.4 Reactome Annotation**

Reactome is an open-source relational database that contains relationships between signaling and metabolic molecules and their organized biological pathways and processes. Pathway information for multiple species is included in the database, and this study only annotates and analyzes pathways for human.

**1.5.5 WikiPathways Annotation**

The WikiPathways database is an open collaborative platform for collecting and disseminating biological pathway models used for data visualization and analysis. This study only annotates and analyzes pathways for human.

**1.6 Screening for Differential Proteins**

The fold change (FC) is determined by the ratio of the mean relative quantitative values of proteins in two sample sets, utilizing three or more replicates. The FC of the protein is calculated between a normal sample and a low-grade undifferentiated spindle cell sarcoma sample. The formula is expressed as follows: R represents the relative quantitative value of the protein, i indicates the sample, and k signifies the protein.

FCA/B,k = Mean (Rik, i∈A)/Mean (Rik, i∈B)

The relative quantitative values of the proteins in the two sample groups were analyzed using a T-test, and the resulting P-value was computed to assess significance, with a threshold set at P < 0.05. The test data must conform to the normal distribution required for the t-test. Prior to the test, the relative quantitative values of proteins must undergo Log2 transformation. The formula is presented below:

Pk = T.test (Log2(Rik, i∈A), Log2 (Rik, i∈B))

For two replicates, the formula is defined as follows: R denotes the relative quantitative value of the protein, i represents the sample, and k indicates the protein.

FCA/B,k = Mean (Rik, i∈A)/(Rik, i∈B)

The standardized coefficient of variation (CV) for each protein in the two comparison groups was calculated to assess the significance of the differences, using a default CV threshold of less than 0.1. The calculation of the formula proceeded as follows:

CVk = SD (A1k/B1k, A2k/B2k)/Mean (A1k/B1k, A2k/B2k)

For no duplicates, the formula is calculated as follows: where R denotes the relative quantitative value of the protein and k denotes the protein.

FCA/B,k = RAk/RBk

In the analysis of variance presented, a differential expression changes exceeding 1.5 was established as the threshold for significant up-regulation, while a change of less than 1/1.5 has been defined as the threshold for significant down-regulation, based on a P value of less than 0.05.

Add criteria for presence or absence: a comparison group of low-grade undifferentiated spindle cell sarcomas (USCSs) and corresponding normal tissues, with FC for low-grade USCS presence and normal tissue absence set to 1000; FC for low-grade USCS absence and normal tissue presence set to 0.001, and both statistical significance P value and CV set to 0.001.

Based on the information in two columns, we screened the presence or absence of protein expression, respectively (1) Found in Sample column with the value of Not Found is regarded as no, which means that it is not identified in the corresponding sample, and the value of High or PeakFound is regarded as yes, which means that it is identified in the corresponding sample; (2) Abundances (grouped) with the value of empty is regarded as no, which means that it is not quantifiable in the corresponding sample, and those without empty is regarded as yes, which means that it is quantifiable in the corresponding sample. (3) Abundances (grouped) is regarded as none, indicating that it is not quantifiable in the corresponding samples, and the value of not empty is regarded as yes, indicating that it is quantifiable in the corresponding samples. The combination of the two conditions of (1) and (2) will be screened for a single group of samples in accordance with the following sample number settings, and the presence or absence of proteins corresponding to each comparative group under the respective conditions will be obtained: 1). one repetition, with a 1 and without a 0; 2). two repetitions, with for 2, without for 0; 3). three repetitions, with for 2, without for 0; 4). four repetitions or more samples, with for 50.0% sample size rounded upwards, without for 0.

**1.7 Functional Enrichment**

We used Fisher's exact test to analyze the significance of the functional enrichment of differentially expressed proteins, using the identified protein as the background. Functional terms with fold enrichment >1.5 and *P* values <0.05 were considered significant.

Enrichment-based Clustering

We use cluster analysis based on protein functional enrichment of differentially expressed proteins (or different fold changes of differentially expressed proteins) to investigate how they might be linked and how their functions might be different (GO, KEGG pathways, Protein domains, Reactome, WikiPathways). First, collect the functional enrichment results of all protein groups, then filter out functions that are significantly enriched (P < 0.05) in at least one protein group. The filtered P value data matrix performs a -Log10 transformation, then followed by one-sided clustering analysis using hierarchical clustering (Euclidean distance, average linkage clustering) on the transformed dataset. The clustering relationships are shown through a heatmap developed by the Heatmap function in the R package ComplexHeatmap.

**1.8 Protein-protein Interaction Network**

All differentially expressed protein database accessions or sequences were searched against the STRING database for protein-protein interactions. We selected only interactions between the proteins in the searched data set, thereby excluding external candidates. We obtained all interactions with a confidence score greater than 0.7 (high confidence), as defined by STRING's "confidence score" metric. The R package "visNetwork" was used to show the STRING interaction network.

**1.9 DrugBank drug target prediction**

We query biomarker drug target information using Drugbank data. Initially, we converted the biomarkers significantly associated with the disease identified through proteomic screening into Uniprot protein names. Subsequently, we acquired the corresponding DrugBank (v5.1.8) (Knox et al., 2010) drug names, types, and FDA approvals for these biomarkers in large quantities.

**References**

Knox, C., Law, V., Jewison, T., Liu, P., Ly, S., Frolkis, A., et al. (2010). DrugBank 3.0: a comprehensive resource for ‘Omics’ research on drugs. Nucleic Acids Research 39(suppl_1), D1035-D1041. doi: 10.1093/nar/gkq1126.

# Supplementary Figures and Tables

## Supplementary Figures


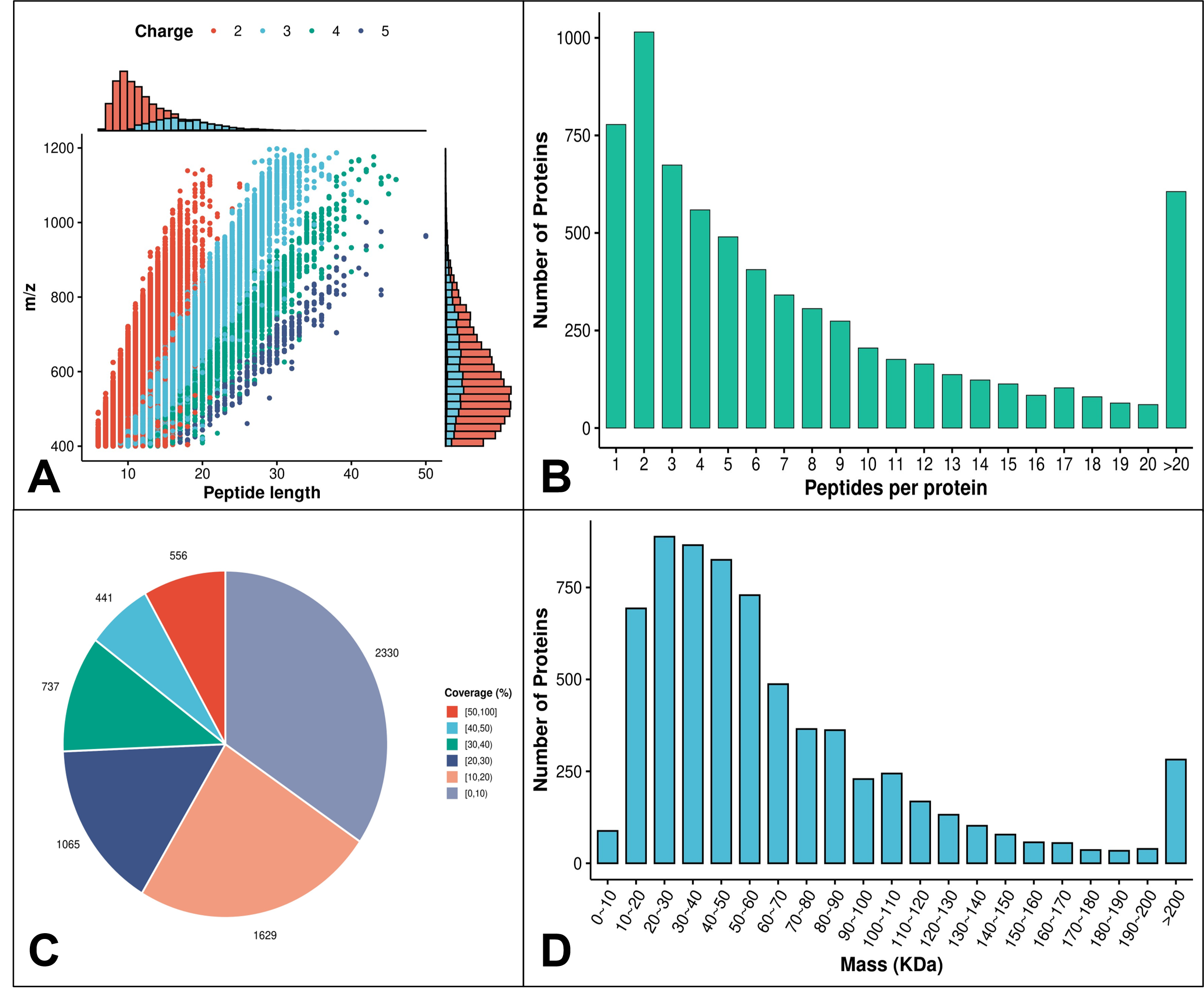


**Supplementary Figure 1.** Data quality control assessments of mass spectrometry downlink. (A) Distribution map of peptide lengths. Most peptides exhibited 2-3 charges, with a distribution of 7-20 amino acids, aligning with established patterns observed in enzymatic and mass spectrometry fragmentation methods. The distribution of peptide lengths identified through mass spectrometry satisfies the quality control criteria. (B) Distribution map of the number of peptide segments. Most proteins are associated with multiple peptides. In quantification, a protein that binds to multiple specific peptides segments or corresponds to multiple spectra enhances the accuracy and confidence of the quantification results. (C) Protein coverage distribution map. Most proteins have less than 30% coverage. (D) Protein molecular weight distribution graph. The molecular weights of the identified proteins were present at different stages and were evenly distributed, indicating that the experimental process did not result in the loss of proteins in certain molecular weight ranges.

**Supplementary Figure 2.** Sample Repeatability Analysis. (A) The Pearson correlation coefficient (PCC) for two samples is determined using the intensity values of all samples, resulting in the creation of a visual heat map. This coefficient measures the correlation degree between the two data sets. A redder color indicates a Pearson's correlation coefficient closer to 1, signifying a stronger correlation between the two samples. (B) Principal Component Analysis (PCA) was performed based on the relative quantitative values of all samples, and visualized PCA plots were drawn. The horizontal and vertical axes show the degree of explanation of PC1 and PC2, with larger values having higher degrees of explanation. The degree of clustering within a group represents how well the grouped samples are reproduced, with duplicates in each group tending to cluster together. (C) The relative standard deviation (RSD) determined from the relative quantitative values of duplicate samples in each group is represented in a box-line graph. The horizontal axis indicates the group names, the vertical axis indicates the RSD values, and different colors represent the various subgroups. A lower overall RSD value reveals increased quantitative repeatability.


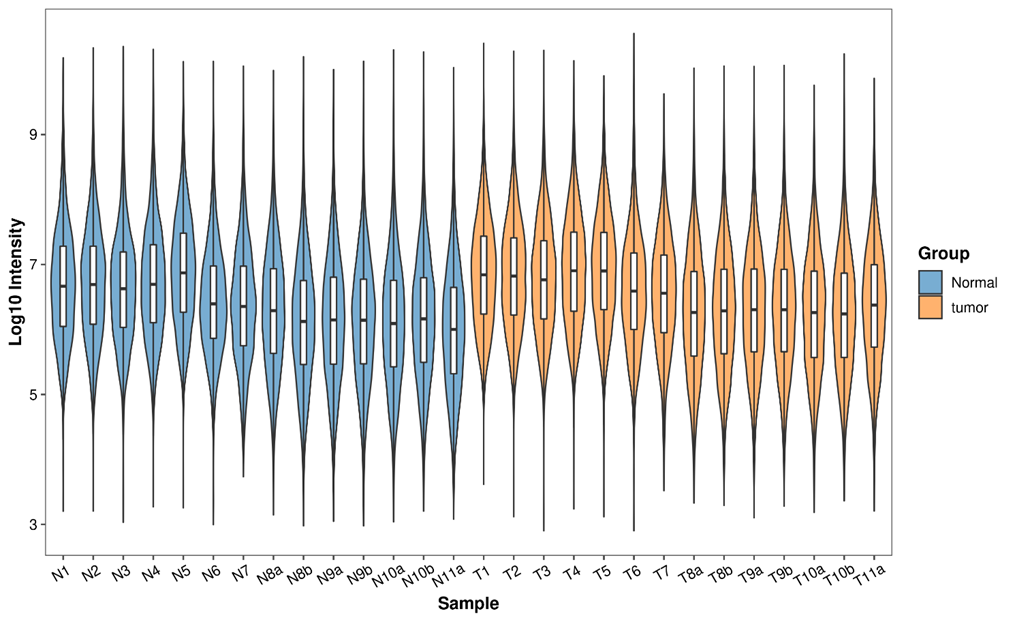


**Supplementary Figure 3.** The horizontal axis represents the sample names, while the vertical axis indicates intensity values transformed using Log10. The color of the violin plot signifies different subgroups. The inner section of the violin plot features a box-and-line representation, where the box illustrates the central 50% of the data distribution within the group. The outer section corresponds to the kernel density plot; a larger area in this region suggests a higher probability of the associated value's distribution. Side-by-side comparisons facilitate an overview of data dispersion both within and between groups, with mean values across samples in the group being approximately equivalent, suggesting high sample quality.


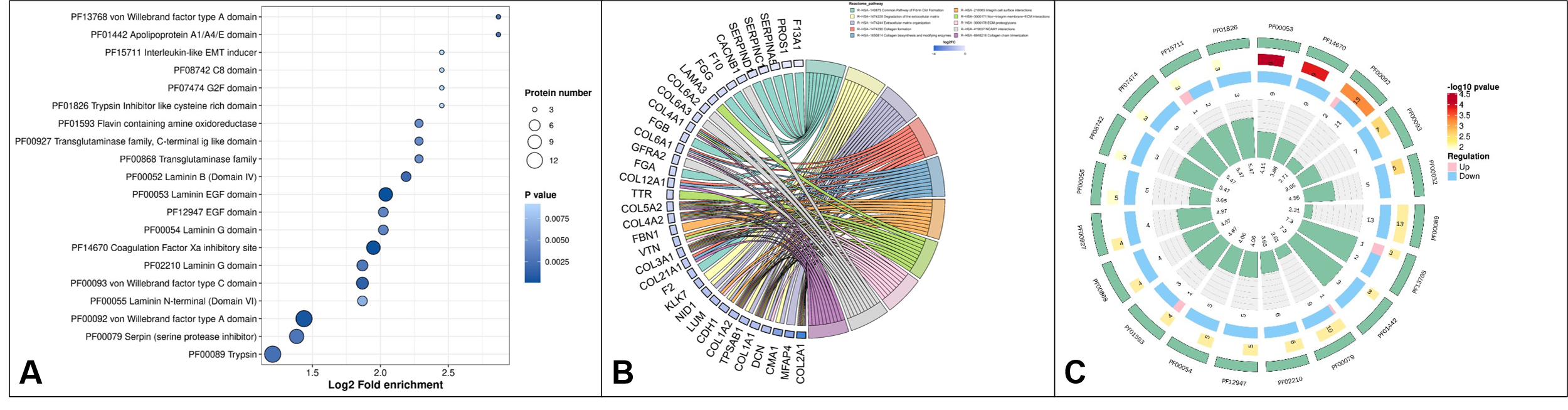


**Supplementary Figure 4.** Protein domain enrichment analysis. (A) Protein domain significantly enriched bubble map, the top enriched protein domain in low-grade USCSs was the laminin EGF domain. (B) Significant enrichment of the chord diagram in KEGG. The proteins identified as enriched in the protein domain include LAMC1, AGRN, LAMA4, HSPG2, LAMA5, LAMA2, LAMB2, LAMA3, and MEGF6. (C) Significant enrichment of the circos plot in protein domain. The two protein domains with the highest enrichment for differential proteins were PF00089 and PF00092.


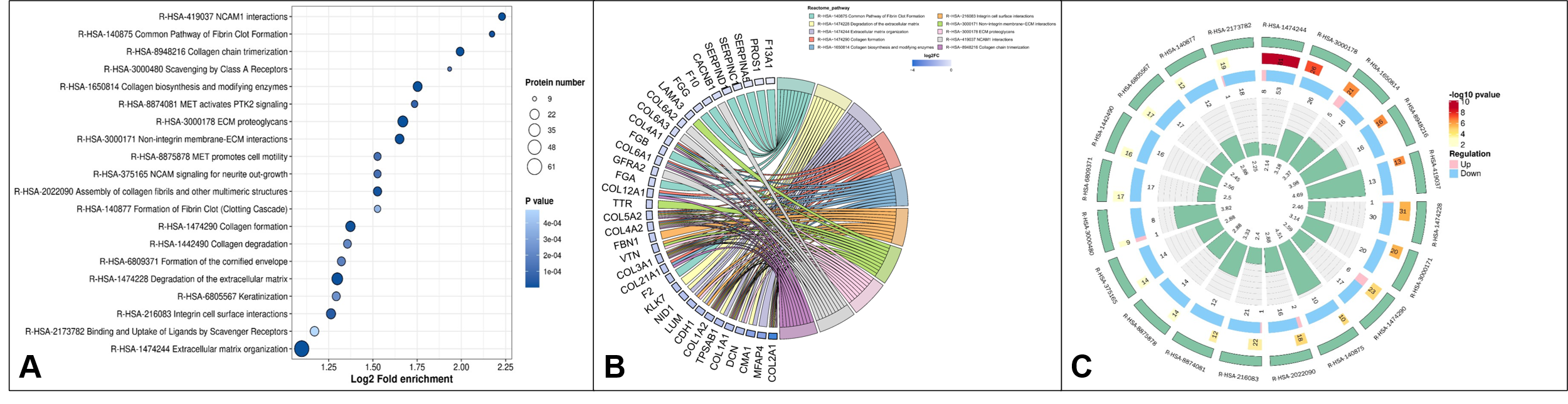


**Supplementary Figure 5.** Reactome pathway enrichment analysis. (A) Reactome pathway significantly enriched bubble map, the top enriched pathway in low-grade USCSs was the extracellular matrix organization. (B) Significant enrichment of the chord diagram in reactome pathway. The proteins identified as enriched in the protein domain include MFAP4, CMA1, DCN, TPSAB1, CDH1, LUM, and NZD1. (C) Significant enrichment of the circos plot in Reactome. The highest differential protein enrichment pathway was R-HAS-1474244.


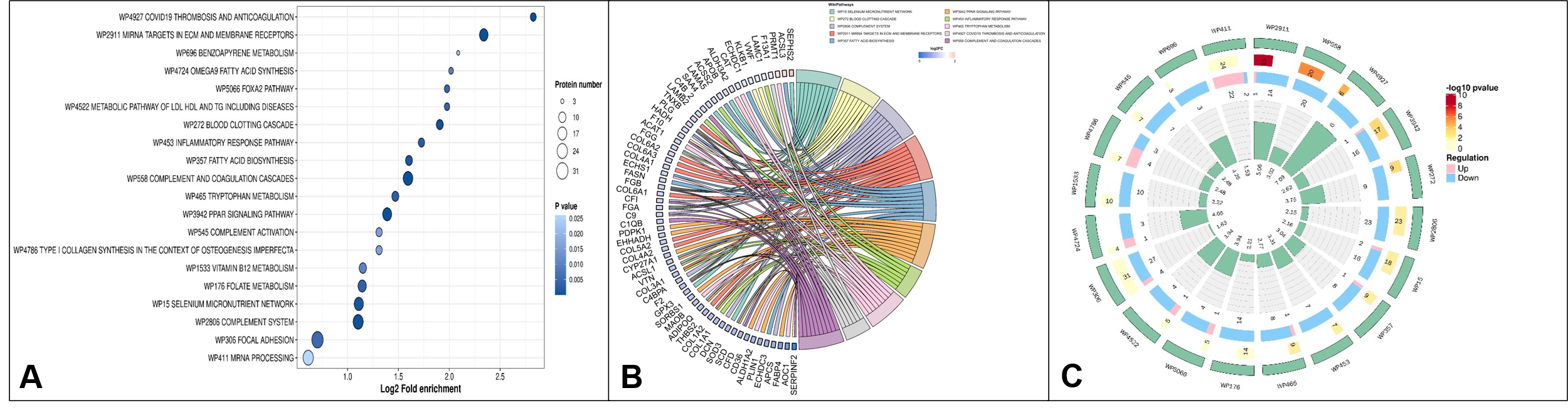


**Supplementary Figure 6.** WikiPathways enrichment analysis. (A) WikiPathways significantly enriched bubble map, the top enriched pathway in low-grade USCSs was the miRNA targets in extracellular matrix and membrane receptors. (B) Significant enrichment of the chord diagram in WikiPathways. The proteins identified as enriched in the pathway include TNXB, COL6A2, COL6A3, COL4A1, COL6A1, COL5A2, COL4A2, COL3A1, THBS2, and COL1A2. (C) Significant enrichment of the circos plot in WikiPathways. The highest differential protein enrichment pathway was WP306.


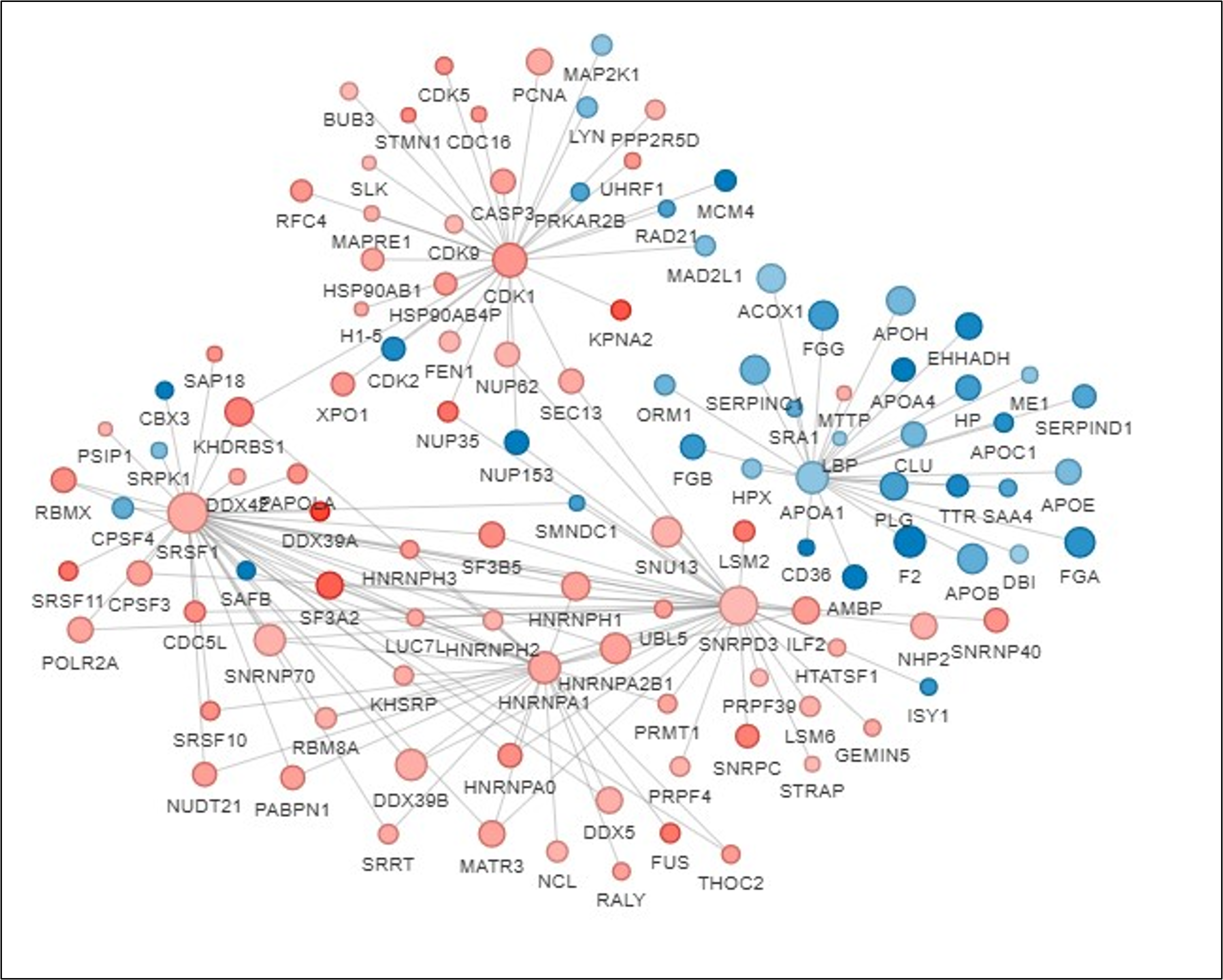


**Supplementary Figure 7.** Protein Interaction Network Analysis. We identified the five proteins with the most significant interactions and delineated their interaction networks. The five most prominent proteins identified were CDK1, LBP, SNRPD3, HNRNPA2B1, and DRSF1. The circles in the figure denote differential proteins, with varying colors indicating differential expression (blue for down-regulated proteins and red for up-regulated proteins). The intensity of the color correlates with the scale of the fold difference, while the size of each circle reflects the number of interacting proteins.
